# The Dawn of Precision Medicine in Fibrotic Interstitial Lung Disease

**DOI:** 10.1016/j.chest.2024.10.042

**Published:** 2024-11-08

**Authors:** Theodoros Karampitsakos, Bochra Tourki, Jose D. Herazo-Maya

**Affiliations:** Division of Pulmonary, Critical Care and Sleep Medicine, Ubben Center for Pulmonary Fibrosis Research, Department of Internal Medicine, Morsani College of Medicine, University of South Florida, Tampa, FL

**Keywords:** fibrotic interstitial lung disease, personalized medicine, precision medicine, prognosis, theragnostic biomarkers

## Abstract

**Topic Importance:**

Interstitial lung diseases (ILDs) represent a broad group of heterogeneous parenchymal lung diseases. Some ILDs progress, causing architectural distortion and pulmonary fibrosis, and thus are called fibrotic ILDs. Recent studies have shown a beneficial effect of antifibrotic therapy in fibrotic ILDs other than idiopathic pulmonary fibrosis (IPF) that manifest progressive pulmonary fibrosis (PPF). However, it remains challenging to predict which patients with fibrotic ILDs will demonstrate PPF. Precision medicine approaches could identify patients at risk for progression and guide treatment in patients with IPF or PPF.

**Review Findings:**

Multiple biomarkers able to highlight disease susceptibility risk, to provide an accurate diagnosis, and to prognosticate or assess treatment response have been identified. Advances in precision medicine led to the identification of endotypes that could discriminate patients with different fibrotic ILDs or patients with different disease courses. Importantly, recent studies have shown that particular compounds were efficacious only in particular endotypes. The aforementioned findings are promising. However, implementation in clinical practice remains an unmet need.

**Summary:**

Substantial progress has been observed in the context of precision medicine approaches in fibrotic ILDs in recent years. Nonetheless, infrastructure, financial, regulatory, and ethical challenges remain before precision medicine can be implemented in clinical practice. Overcoming such barriers and moving from a one-size-fits-all approach to patient-centered care could improve patient quality of life and survival substantially.

Interstitial lung diseases (ILDs) represent a heterogeneous group of > 200 parenchymal lung diseases. A substantial proportion of ILDs such as idiopathic pulmonary fibrosis (IPF), connective tissue disease-associated ILD (CTD-ILD), fibrotic hypersensitivity pneumonitis (fHP), and fibrotic sarcoidosis are associated with radiologic fibrosis and thus are called fibrotic ILDs.^1^ Evidence has shown a beneficial effect of antifibrotic therapy in fibrotic ILDs other than IPF that manifest as progressive pulmonary fibrosis (PPF).^1^ While we are heading toward a more uniform approach regarding the use of antifibrotic therapy for these patients, management based on endotypes remains an unmet need.^1^^,^^2^

The dawn of precision medicine could address this unmet need. According to the US Food and Drug Administration, precision medicine can be defined as “an innovative approach aiming to tailor disease prevention and treatment which takes into account differences in people’s genes, environments, and lifestyles. The goal of precision medicine is to target the right treatment to the right patients at the right time.”^3^ Based on the aforementioned definition, precision medicine includes: (1) biomarkers able (a) to identify patients at risk for a disease timely, (b) to diagnose particular disease entities, (c) to predict which of the patients with these disease entities are at risk of progression, and (d) to predict and assess treatment response and (2) treatment regimens that could be beneficial for a subset of patients with a specific phenotype to reduce unnecessary exposure to ineffective drugs and side effects.^2^

Precision medicine already has been implemented in other diseases including lung cancer and asthma.^2^ In the context of fibrotic ILDs, although substantial research progress has been made, little progress has been made regarding the implementation of these findings in clinical practice. In this review, we present the progress of precision medicine in fibrotic ILDs and include articles able to contribute to precision medicine based on the definitions noted. We focus on the most common ILDs associated with radiologic fibrosis based on the classification in the most recent joint American Thoracic Society, European Respiratory Society, Japanese Respiratory Society, and Asociación Latinoamericana de Tórax clinical practice guidelines.^1^ Finally, we present limitations and challenges that need to be addressed for the implementation of precision medicine in everyday clinical practice.

## Literature Search

To supplement prior knowledge of this topic, we searched PubMed for articles related to precision medicine in fibrotic ILDs, using the terms *precision medicine*, *personalized medicine*, *disease susceptibility*, *diagnosis*, *prognosis*, *treatment response*, *biomarkers*, *theragnostic biomarkers*, *fibrotic interstitial lung diseases*, *IPF*, *HP*, *CTD-ILD*, *sarcoidosis associated pulmonary fibrosis*, and *PPF* from January 1, 2004, through August 1, 2024. We focused mainly on recent articles. Titles and abstracts were reviewed for scope, and the full text of pertinent articles were reviewed for thematic content.

## Evidence Review

### Disease Susceptibility

#### Familial Pulmonary Fibrosis

It has been established that genetic factors can contribute to the risk of familial pulmonary fibrosis (FPF) development. Thus, the potential of genetic screening for various mutations has been investigated in an effort to provide those patients with a diagnosis in a timely fashion. Surfactant protein mutations mostly have been linked to FPF development.^4^ Because of the high penetrance of mutations related to surfactant homeostasis, the predictive power for disease development after genetic sequencing is high.^5^ Telomerase complex mutations seem to be more frequent in FPF than IPF, but are probably not disease specific. They are transmitted mainly in an autosomal-dominant manner and have been linked to incomplete penetrance.^5^^,^^6^ The absence of telomerase complex mutations does not exclude the risk of disease resulting from other inherited telomere abnormalities. Moreover, presence of telomere shortening, which is a common finding in FPF, does not necessarily mean presence of mutations, yet it is suggestive.^7^ Finally, *ELMOD2*, a gene that is expressed in type II alveolar epithelial cells and alveolar macrophages, also has been associated with pulmonary fibrosis susceptibility in Finland.^8^ Based on all the aforementioned, the European Respiratory Society published a statement defining FPF as “any fibrotic ILD occurring in at least 2 blood relatives that are first- or second-degree family members” and supported offering genetic sequencing according to national directives or legislation for families with proven monogenic disease.^5^

#### Idiopathic Pulmonary Fibrosis

Polymorphisms mainly associated with host defense, telomere length, surfactant biogenesis, and cellular mitogenesis have been reported in patients with IPF.^2^^,^^9^ The polymorphism rs35705950 in the promoter region of *MUC5B* is the one with the strongest link to IPF susceptibility, probably because of its role in mucosal host defense.^10^^,^^11^ The effect of this polymorphism might be stronger in male patients.^12^ *MUC5AC* also has been linked to disease susceptibility, further highlighting the role of mucins in IPF. Different variants in *TOLLIP* reportedly have opposite effects on the risk of pulmonary fibrosis development. Multiple other polymorphisms in genes including *TGFβ-1*, *IL1RN*, *IL8*, and *HLA DRB1∗1501* might represent risk factors for IPF development; yet, data are still not that strong (as in the case of *MUC5B*) and need to be replicated.^2^ A meta-analysis including a large number of patients and control participants identified 7 novel IPF loci and suggested differences in the genetic background around the world.^12^ Large genome-wide association studies and subsequent meta-analyses have provided robust data for at least 20 common single nucleotide polymorphisms (SNPs) linked to IPF with minor allele frequency of > 5%, further corroborating the association between IPF susceptibility and impaired host defense, mammalian target of rapamycin signaling, cell-to-cell adhesion, and telomere maintenance.^2^^,^^11^^,^^13^ The role of telomere maintenance also is highlighted by the fact that short telomere length has been described in patients with IPF compared with age-matched control participants and likely has a role in the age of disease onset.^14^ Finally, rare protein-altering variants in *TERT*, *TERC*, *PARN*, and *RTEL1* are more frequent in patients with IPF compared with control participants. Interestingly, patients without the *MUC5B* rs35705950 polymorphism are more likely to have the *TERT* variant.^15^

#### CTD-ILD, Fibrotic Hypersensitivity Pneumonitis, and Other Rare ILDs

**Figure 1:**
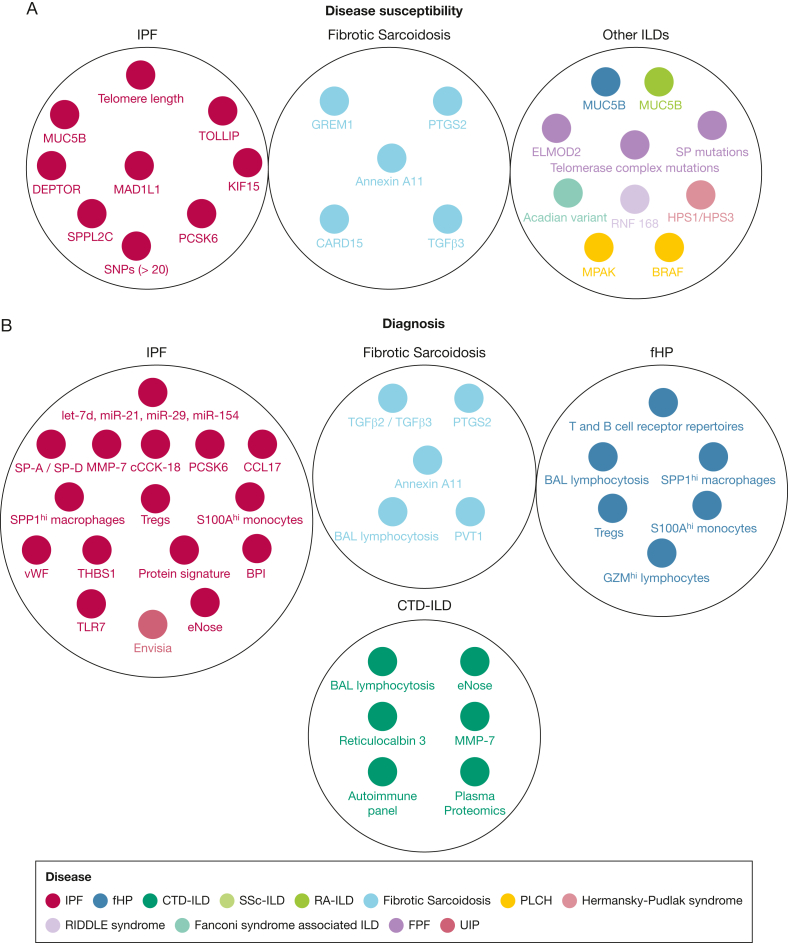
A, B, Diagrams showing the main biomarkers able to predict disease susceptibility (A) and diagnosis (B) in patients with fibrotic ILD. Presence of the same biomarkers in multiple diseases highlights common pathways associated with different fibrotic ILDs. Except diseases, we present a different color for UIP, given that Envisia Genomic Classifier can only suggest presence of UIP and not necessarily presence of a particular disease. BPI = bactericidal permeability-increasing protein; CTD-ILD = connective tissue disease-associated interstitial lung disease; fHP = fibrotic hypersensitivity pneumonitis; FPF = familial pulmonary fibrosis; ILD = interstitial lung disease; IPF = idiopathic pulmonary fibrosis; PLCH = pulmonary Langerhans cell histiocytosis; PPF = progressive pulmonary fibrosis; RA-ILD = rheumatoid arthritis-associated interstitial lung disease; RIDDLE = radiosensitivity, immunodeficiency, dysmorphic features and learning difficulties; SP = surfactant protein; SSc-ILD = systemic sclerosis associated interstitial lung disease; UIP = usual interstitial pneumonia.

The polymorphism rs35705950 has been associated with rheumatoid arthritis-usual interstitial pneumonia, suggesting that some pathways such as those related to host defense are common among patients with usual interstitial pneumonia (UIP) of different causes.^16^ Findings suggest that the same *MUC5B* polymorphism and other common variants in IPF such as *TERC*, *DSP*, and *IVD*, are relevant in fHP, which implies commonalities in the cause of these fibrotic diseases.^9^^,^^17^ Multiple other mutations have been associated with some rare, and potentially fibrotic, ILDs. For example, mutations in *MAPK* and *BRAF* have been linked to pulmonary Langerhans cell histiocytosis, and mutations in *HPS1* or *HPS3* have been linked to Hermansky-Pudlak syndrome. Finally, mutations in the *RNF168* gene represent a risk factor for RIDDLE syndrome, and the Acadian variant has been described in Fanconi syndrome associated-ILD.^18^, ^19^, ^20^ Figure 1A summarizes disease susceptibility variants identified in fibrotic ILDs.

#### Sarcoidosis-Associated Pulmonary Fibrosis

Sarcoidosis-associated pulmonary fibrosis is not necessarily an end-stage manifestation of inflammatory sarcoidosis, but might be a distinct process sharing similarities with other fibrotic ILDs, including fHP.^21^ SNPs encoding *GREM1*, a gene directly related to tissue repair, *TGFβ3*, and *CARD15* have been associated with susceptibility to fibrotic sarcoidosis.^22^, ^23^, ^24^ The -765G→C polymorphism in *PTGS2*, a key regulatory enzyme for the synthesis of the antifibrotic prostaglandin E(2), has been associated with susceptibility to fibrotic sarcoidosis.^25^ Importantly, some SNPs of Annexin A11 (rs1049550, rs12779955), a calcium-dependent membrane-binding protein with an established role in the pathogenesis of sarcoidosis, have been associated with increased susceptibility to fibrotic sarcoidosis.^26^

### Diagnosis

#### Idiopathic Pulmonary Fibrosis

Despite the widespread use of high-resolution CT imaging and the implementation of multidisciplinary discussion after meticulous investigation, diagnosis of IPF often is challenging.^1^ Given that precision matters, biomarkers could contribute to the accuracy of diagnosis. Plasma matrix metalloproteinase (MMP) 7 of > 1.75 ng/mL, surfactant protein D (SP-D) of > 31 ng/mL, and osteopontin of > 6 ng/mL differentiated patients with IPF from patients with other idiopathic interstitial pneumonias, but not from patients with rheumatoid arthritis-associated ILD (RA-ILD).^27^ C-C motif chemokine ligand 17, the glycoproteins thrombospondin 1, von Willebrand factor, and bactericidal permeability-increasing protein have shown potential to differentiate patients with IPF from control participants.^28^ This further supports the concept that abnormal coagulation is a key mechanism in the pathogenesis of pulmonary fibrosis. The combination of peripheral blood proteins as a way to diagnose IPF also has been used previously. For example, a protein signature including MMP-1, MMP-7, MMP-8, tumor necrosis factor receptor superfamily member 1A, and insulin-like growth factor binding protein 1 was able to discriminate control participants from patients with IPF with a specificity of 98.1% and a sensitivity of 98.6%.^29^

Epithelial cell markers such as circulating caspase-cleaved cytokeratin 18 seemed to be higher in patients with IPF than control participants.^30^ Serum levels of epithelium-derived proteins surfactant protein A (SP-A) and C-pro-SP-B were lower in patients with other ILDs than in patients with IPF. Several other biomarkers, including decreased miRNAs (ie, miR-29, let-7d) or increased microRNAs (miR-21 and miR-154) have been shown to have diagnostic potential in IPF, but this has not been implemented in clinical practice.^2^

Finally, in recent years, interest in the transcriptome of fibrotic lung tissue, which is the predominant site of the ILDs, has increased. Genomic analysis of tissue derived from transbronchial biopsies led to the Envisia Genomic Classifier (Envisia Genomic Classifier [Veracyte]), a commercially available genomic classifier with high specificity for histopathologic UIP pattern.^31^ However, a limitation is that genomic UIP does not necessarily mean IPF, and thus the possibility of other fibrotic ILDs cannot be excluded. The most promising noninvasive diagnostic tool with potential to discriminate different fibrotic ILDs is eNose, a method able to analyze volatile organic compounds in exhaled breath.^32^ A combination of Envisia Genomic Classifier and eNose might be the key to discriminate fibrotic ILDs in the future with less interventional techniques, thus avoiding the adverse events related to surgical lung biopsy (Fig 1B).

#### Fibrotic Hypersensitivity Pneumonitis

Bronchoalveolar lavage (BAL) lymphocytosis has been widely considered one of the most reliable and clinically applicable markers able to diagnose fHP over IPF. However, even this marker has limitations, given that it cannot discriminate fHP from other fibrotic ILDs such as CTD-ILD.^1^ A major step for the accurate diagnosis of hypersensitivity pneumonitis was the recently published study that demonstrated single-cell immune aberrations in peripheral blood mononuclear cells and BAL from patients with FHP.^33^ In this work, S100A^hi^ monocytes and SPP1^hi^ macrophages were common in both IPF and fHP. However, GZM^hi^ T lymphocytes represented a unique finding in fHP.^33^ Given that this pattern of BAL lymphocytosis seems to be unique in fHP and given that this was a high-quality study including patients from different countries (United States and Mexico), GZM^hi^ T lymphocytes in BAL might be the key to diagnose fHP accurately in the future.^33^ Specific microbiome patterns in BAL and immune response-related genes in lung tissue also could contribute to the diagnosis of fHP vs IPF.^34^ Genetic studies in patients with fibrotic ILDs including fHP identified mutations in *TERT*, *TERC*, *RTEL1*, and *PARN*, with *TERC* mutations being associated with younger age of diagnosis.^35^ Finally, plasma biomarkers related to aging and the fibrotic process per se hold also promise mainly for the discrimination of fibrotic and nonfibrotic hypersensitivity pneumonitis. For example, plasma levels of growth differentiation factor 15, a protein of the transforming growth factor beta (TGFβ) superfamily, were able to differentiate fHP from nonfibrotic hypersensitivity pneumonitis.^36^

#### CTD-ILD

Given the presence of established autoimmune panels able to lead to the diagnosis of CTD-ILD along with the clinical and radiographic findings, the research effort for novel biomarkers seems to be less extensive. Only recently, machine learning of plasma proteomics consistently distinguished CTD-ILD from IPF.^37^ Other studies focused on biomarkers that showed promising findings in IPF. For example, MMP-7 has been suggested as a marker of ILD in patients with rheumatoid arthritis and systemic sclerosis.^38^^,^^39^ Of note, a well-designed study including patients with IPF, RA-ILD, and other ILDs showed that among patients with increased MMP-7, the absence of concomitant rise in SP-D and osteopontin was indicative of a non-IPF or non-UIP ILD.^27^ Moreover, a marker of alveolar epithelial function named reticulocalbin 3 was higher in patients with CTD-ILDs than in patients with IPF, and thus holds promise; yet, further studies are needed.^40^

#### Sarcoidosis-Associated Pulmonary Fibrosis

Specific TGFβ SNPs, as well as polymorphisms in TGF-β2 and TGF-β3, have been observed in patients with sarcoidosis-associated pulmonary fibrosis,^23^^,^^41^ yet larger studies are needed to validate this finding. SNPs at *PTGS2* also have been associated with fibrotic sarcoidosis,^25^ whereas a recent genome-wide association study investigating patients with fibrotic sarcoidosis of African descent identified relevant variants (rs115529936, rs74730278, rs115311148, and rs115809470) in *PVT1*, a long noncoding RNA known for its role in lymphomas, other malignancies, and angiogenesis.^42^

### Prognosis

#### Idiopathic Pulmonary Fibrosis

**Figure 2:**
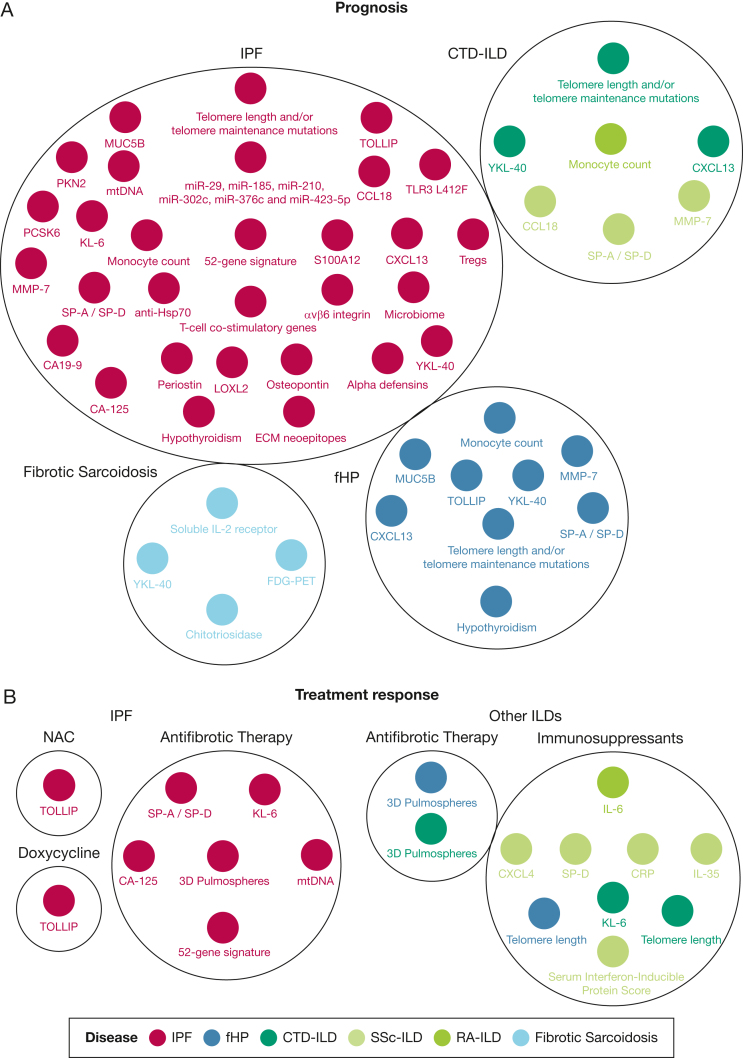
A, B, Diagrams showing the main biomarkers able to indicate prognosis (A) or to assess treatment response (B) in patients with fibrotic ILD. Presence of the same biomarkers in multiple diseases highlights common pathways associated with different fibrotic ILDs. CRP = c-reactive protein; CTD-ILD = connective tissue disease-associated interstitial lung disease; fHP = fibrotic hypersensitivity pneumonitis; ILD = interstitial lung disease; IPF = idiopathic pulmonary fibrosis; NAC = N-acetylcysteine; RA-ILD = rheumatoid arthritis-associated interstitial lung disease; SSc-ILD = systemic sclerosis associated interstitial lung disease.

Gene polymorphisms, microRNAs, and telomere abnormalities have been suggested as predictors of mortality in patients with IPF. Figure 2A provides a summary of the prognostic biomarkers for fibrotic ILDs including IPF. The same *MUC5B* polymorphism (rs35705950) associated with IPF susceptibility also has been associated with decreased mortality in IPF^10^^,^^43^; yet, this requires further investigation given that other reports suggested that the prognostic accuracy might be a source of index event bias.^2^^,^^44^ Regarding other SNPs, a functional variant of *TOLLIP*, rs5743890, has been linked to increased mortality in IPF.^45^ A toll-like receptor 3 functional variant (Leu412Phe, toll-like receptor 3 L412F) and a variant in protein kinase N2 (rs115982800) also seem to be indicators of disease progression in patients with IPF,^46^^,^^47^ whereas recently, a genome-wide association study identified a novel variant in the calcium-dependent serine endoprotease *PCSK6* that was associated with IPF survival and reached genome-wide significance.^48^

In addition to gene variants, microRNAs such as miR-185, miR-210, miR-302c, miR-376c, and miR-423-5p were higher in the lung tissue of patients with IPF characterized as rapid progressors than in the tissue of patients characterized as slow progressors.^49^ Regarding peripheral blood, lower miR-29 expression in plasma and serum was linked to higher mortality in 2 cohorts with IPF.^50^ In the context of telomeres, shorter leucocyte telomere length has been linked to worse outcomes in IPF.^51^ Given the high quality of the data, a result indicating telomere length below the 10th percentile might affect patients' management in some centers of expertise. Moreover, mutations in genes associated with telomere maintenance such as *TERT*, *TERC*, *PARN* and *RTEL1* seem to be markers of worse outcomes.^35^

In addition to genetic and epigenetic variations, changes in gene expression, particularly in peripheral blood, also have been shown to predict mortality in IPF.^52^, ^53^, ^54^ Α 52-gene signature in peripheral blood predicted outcomes in 6 independent cohorts with IPF.^55^ Cellular deconvolution of the upregulated genes of this signature showed that their cellular source mainly was monocytes. This fueled several studies showing that increased monocyte count is an indicator of poor outcomes in IPF.^2^^,^^56^ Recently, a single-cell immune atlas in IPF demonstrated an outcome-predictive increase in classical monocytes and T regulatory cells.^54^ Further investigation showed that CD14^+^CD163^–^HLA-DR^low^ monocytes are the subpopulation of monocytes that drives mortality in IPF. In particular, a 230-gene profile derived from this immune subpopulation and a subset of 13 genes from the same profile were able to discriminate patients with increased risk of poor outcomes. Results were reproducible in peripheral blood, BAL, and lung tissue.^57^ CD14^+^CD163^–^HLA-DR^low^ monocytes seem to be associated with T-cell exhaustion, expansion of Tregs, and suppression of natural killer cells.^57^ This could be the reason that decreased expression of T-cell costimulatory genes, expansion of Tregs, and suppression of natural killer cells have been associated with IPF progression.^54^^,^^55^^,^^57^, ^58^, ^59^ These findings coupled with those of multiple other studies showing that biomarkers of immune dysregulation such as serum levels of chemokine (C-X-C motif) ligand 13 (CXCL13) and chemokine ligand 18 (CCL18) can predict pulmonary fibrosis progression reliably.^60^^,^^61^

Altered microbiome also seems to have a cardinal role for immune dysregulation in IPF, given that increased bacterial burden and particular pathogens in BAL predicted lung function decline and survival.^62^ The altered microbiome could be a plausible explanation for the reason that the antimicrobial peptides named alpha defensins, which are identified mainly in alveolar type II cells and represent a cardinal element of innate immunity, have shown potential in the prognostication of acute exacerbation in patients with IPF.^63^

Given the emerging evidence suggesting that epithelial cell injury is a driver of pulmonary fibrosis progression and perhaps of immune dysregulation, the prognostic potential of multiple markers reflective of alveolar epithelial cell injury has been investigated in fibrotic ILDs. The Prospective Observation of Fibrosis in the Lung Clinical Endpoints Study (PROFILE) study investigated epithelium-derived proteins in IPF and demonstrated that increased values of 4 serum biomarkers (SP-D, CA19-9, CA-125, and MMP-7) consistently were predictive of poor outcomes.^64^ These findings coupled with other studies demonstrating that higher SP-D, SP-A, MMP-7, and other MMPs are negative prognosticators in IPF.^2^^,^^64^ Increased levels of markers reflective of epithelial injury such as osteopontin and Krebs von den Lungen-6 hold promise as biomarkers of acute exacerbations in patients with pulmonary fibrosis.^2^^,^^65^^,^^66^ Specifically KL-6 might be a better marker in acute exacerbations, given that prediction of disease progression or mortality was not reproducible in all studies.^2^

Studies focusing on the metabolic state of alveolar epithelial cells demonstrated interesting and clinically applicable findings. The presence of hypothyroidism was associated with increased mortality in patients with IPF.^67^ Increased mitochondrial DNA consistently was associated with poor survival in IPF and predicted the risk of acute exacerbation.^68^^,^^69^ Extracellular matrix neoepitopes also were linked to disease progression in IPF. In particular, 6 neoepitopes (BGM, C1M, C3A, C3M, C6M, and CRPM) were significantly higher in patients with progressive disease. The strongest association between the 3-month biomarker change and survival was recorded for CRPM.^70^ Finally, several other biomarkers such as YKL-40, periostin, lysyl oxidase-like 2, S100 calcium-binding protein A12, antiheat shock protein 70, baseline levels of type I and III collagen turnover, and ανβ6 integrin have been proposed as prognostic markers in IPF.^2^^,^^55^^,^^71^, ^72^, ^73^, ^74^, ^75^

#### Fibrotic Hypersensitivity Pneumonitis

Studies for the prognostication of fHP focused mainly on markers that reliably predicted outcomes in IPF, given the common core pathways between these two diseases. Polymorphisms and haplotypes of *MUC5B* and *TOLLIP* have been suggested as prognosticators of functional or radiographic status, or both, in fHP, highlighting the presence of commonalities in terms of host defense and immune response between IPF and fHP.^76^, ^77^, ^78^ Genetic analysis of patients with fibrotic ILDs including fHP showed that telomere-related lung fibrosis was heterogeneous diagnostically, but uniformly progressive.^35^^,^^77^ Epigenetic studies in BAL including patients with fHP among other fibrotic ILDs demonstrated that miR-21 and miR-92a were downregulated in patients with PPF compared with patients without PPF.^79^ Meticulous investigation of circulating plasma biomarkers showed that CXCL13, MMP-7, and vascular cell adhesion protein 1 predicted mortality in patients with fHP.^80^ Limited data suggest that epithelial injury markers such as KL-6 and SP-D also might have a prognostic role.^81^ Finally, YKL-40 and hypothyroidism might be markers of functional status and mortality, respectively; however, larger studies are needed to validate this proposal.^82^^,^^83^

#### CTD-ILD

The *MUC5B* polymorphism (rs35705950) associated with IPF and RA-ILD susceptibility did not discriminate outcomes in RA-ILD and systemic sclerosis ILD.^10^^,^^16^^,^^43^^,^^84^ On the contrary, biomarkers related to immunity with a prognostic role in IPF were reproducible in CTD-ILD. Increased monocyte count was a negative prognostic marker in a small study of patients with RA-ILD and in studies that involved multiple fibrotic ILDs including CTD-ILD.^85^ High circulating plasma levels of CXCL13 were predictive of negative outcomes in CTD-ILD, and high CCL18 in serum has been a negative prognostic marker in more than one study of patients with systemic sclerosis ILD.^80^, ^86^, ^87^ Another protein related to innate immune responses after extracellular matrix remodeling that was predictive of outcomes in systemic sclerosis ILD and myositis ILD was YKL-40.^88^ Regarding other biomarkers related to the extracellular matrix, increased MMP-7 has been a validated biomarker in systemic sclerosis ILD.^89^ Finally, given that epithelial injury is relevant in most of the fibrotic ILDs, surfactant proteins including SP-D have been associated with systemic sclerosis ILD progression.^86^

#### Sarcoidosis-Associated Pulmonary Fibrosis

More data are needed for the establishment of reliable prognosticators in sarcoidosis-associated pulmonary fibrosis. To this end and similarly with nonfibrotic sarcoidosis, serum angiotensin converting enzyme, soluble IL-2 receptor, calcium, chitotriosidase, and fludeoxyglucose F18-PET are used to predict progression.^90^^,^^91^ Other markers that have been correlated with disease severity are MMP-12 and YKL-40; however, larger studies are needed to validate these findings.^92^^,^^93^

### Prediction and Assessment of Treatment Response

#### Idiopathic Pulmonary Fibrosis

In recent years, we have witnessed a scientific explosion of biomarkers that are able to predict or assess treatment response in IPF (Fig 2B). *TOLLIP* TT and CC genotypes were associated with better and worse response to N-acetylcysteine, respectively.^94^ This has fueled the design of the Prospective Treatment Efficacy in IPF Using Genotype for Nac Selection (PRECISIONS) trial, which aims to assess N-acetylcysteine response based on *TOLLIP* gene variants in IPF. Most recently, analysis of the CleanUP IPF for the Pulmonary Trials Cooperative (CleanUP-IPF) trial based on *TOLLIP* genotype showed that treatment with doxycycline led to improved survival in patients with a TT genotype, but worse survival in patients with a CC genotype.^95^ This finding highlights the importance of precision medicine, given that initial analysis without consideration of endotypes did not show a benefit from doxycycline use.^95^ Incorporation of testing for *TOLLIP* TT genotype in clinical practice might be worthy based on these results.

Regarding biomarkers able to assess treatment response to antifibrotic compounds, the results of the Biomarkers of Extracellular Matrix Turnover in Patients With Idiopathic Pulmonary Fibrosis Given Nintedanib (INMARK) trial showed that treatment with nintedanib did not affect the rate of change of collagen neoepitopes.^96^ Based on other data, serum SP-D might be a biomarker of pirfenidone effectiveness,^97^ especially if measured serially,^98^ whereas some promising preliminary data have been published for the theragnostic potential of SP-A in relationship to nintedanib and pirfenidone.^99^ The aforementioned findings were promising, yet, validation is required. Another promising finding was that the change in mitochondrial DNA after 3 months of treatment correlated with pirfenidone response after 1 year.^68^ Moreover, the 52-gene signature in peripheral blood also demonstrated potential as a biomarker of treatment response in IPF.^55^ Finally, other studies showed that KL-6, but not CCL18, might be a theragnostic biomarker in IPF.^2^^,^^72^

In the context of invasive biomarkers, 3-dimensional pulmospheres (spheroids derived from lung biopsy) could predict the antifibrotic that is more likely to be beneficial for every patient through a model simulating the lung microenvironment.^100^ However, implementation in clinical practice of noninvasive methods with potential to predict treatment response such as eNose seems to be more feasible.^101^

#### Other Fibrotic ILDs

The most important study for precision medicine in other fibrotic ILDs was the one showing that patients with fHP or unclassifiable ILD and leukocyte telomere length of less than the 10th percentile experienced reduced survival if exposed to mycophenolate mofetil or azathioprine in 2 independent cohorts.^102^ This couples with previous evidence showing that leukocyte telomere length might be a biomarker able to predict detrimental effects of immunosuppression in IPF.^103^ Based on these findings, clinical decisions for immunosuppression guided by telomere length seem to be plausible.

Other biomarkers such as serial values of KL-6, IL-35, SP-D, CXCL-4, C-reactive protein, and the combination of IL-6 and its soluble receptor have been suggested as predictors of lung function decline after implementation of immunosuppressants in CTD-ILDs.^104^, ^105^, ^106^, ^107^ Furthermore, a recent model aiming to predict treatment response to cyclophosphamide and mycophenolate mofetil in systemic sclerosis ILD has been published.^108^ Based on that model, a higher interferon-inducible protein score was predictive of better response to immunosuppression and potentially could identify patients who are most likely to experience benefit from mycophenolate mofetil or cyclophosphamide.^108^ Finally, given the promising findings in IPF, the model of 3D pulmospheres was tested in non-IPF ILDs and retained the accuracy for prediction of antifibrotic response.^109^

## Future Directions

### Limitations and Challenges for the Implementation of Precision Medicine in Clinical Practice

Substantial progress has been observed in terms of biomarkers for patients with fibrotic ILDs. However, these biomarkers can have an impact as part of precision medicine only if they reach clinical practice. To accomplish that goal, a biomarker should provide actionable information that is worth the effort to overcome the challenges for clinical applicability. Such actionable information might be a result able to indicate benefit from a particular treatment, a result guiding to a specific diagnosis, or a result highly indicative of disease activity. Such results could be obtained by randomized controlled trials that take into consideration patient endotypes.^95^ Studies not taking into consideration endotypes should not lead to definite conclusions. A study reporting negative results for a particular compound in the entire population of patients with a specific disease does not exclude the possibility that this compound might be efficacious in a proportion of patients with a specific endotype.^95^

If this first goal is accomplished, then multiple infrastructure, financial, regulatory, and ethical challenges must be overcome.^110^ In terms of infrastructure, a feasible, universal model able to be deployed across the world, and not only to particular centers, is required. Technical variabilities should be minimized so that results could be reproducible. Along with this, a scalable learning system able to help clinicians keep pace with advances is needed. The main financial challenge stems from the fact that these precision medicine approaches are expensive. However, scientists should highlight the potential cost-effectiveness of precision medicine to the political community by showing that it can reduce costs resulting from inappropriate use of expensive treatments and costs from hospitalizations that could be avoided with patient-centered management. In this way, we also could overcome regulatory challenges. Given that precision medicine is vulnerable to the pacing issue associated with the fact that advances quickly outpace laws and regulations, regulatory bodies could expedite related approvals. They also need to couple innovation with robust safety standards so that big data could be used without stigmatizing patients. Finally, another challenge is the holistic approach of patients. This holistic approach should be able to handle the psychological consequences of a laboratory result showing, for example, that a patient is a rapid progressor.^110^

**Figure 3:**
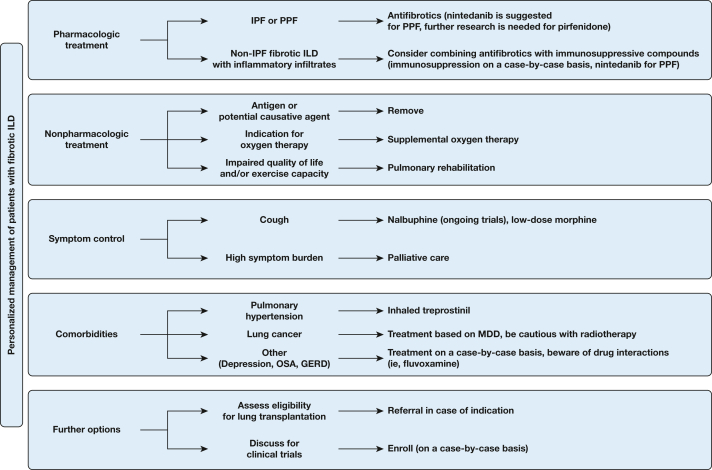
Diagram showing the personalized management of patients with fibrotic ILD toward a patient-oriented approach.GERD = gastroesophageal reflux disease; ILD = interstitial lung disease; IPF = idiopathic pulmonary fibrosis; MDD = multidisciplinary discussion; PPF = progressive pulmonary fibrosis.

Given that all these have not been implemented into clinical practice, personalized management currently involves management based on the clinical subphenotype. The considerable progress toward this direction is summarized in Figure 3. Indeed, management is now personalized and does not include only the prescription of an antifibrotic compound. Compounds for comorbidities, such as inhaled treprostinil for pulmonary hypertension, or compounds for chronic cough, like nalbuphine or low-dose morphine, seem to be very promising. While seeking precision through molecular endotypes, clinicians should not forget that precision in clinical subphenotypes also matters.^2^^,^^110^

## Summary

Considerable progress has been observed regarding personalized medicine approaches in fibrotic ILDs in recent years. However, infrastructure, financial, regulatory, and ethical challenges remain to be overcome for the implementation of precision medicine in clinical practice. Overcoming such barriers and moving from a one-size fits all approach to patient-centered care could improve patient quality of life and survival substantially.

## Funding/Support

This study was supported by the USF Foundation Ubben Family Fund and the Ubben Center for Pulmonary Fibrosis Research.

## Financial/Nonfinancial Disclosures

None declared.
